# Pulsed Laser Deposition of Halide Perovskites with over 10-Fold Enhanced Deposition Rates

**DOI:** 10.1021/acs.jpclett.5c00047

**Published:** 2025-01-31

**Authors:** Vojta Kliner, Tatiana Soto-Montero, Jasmeen Nespoli, Tom J. Savenije, Martin Ledinský, Monica Morales-Masis

**Affiliations:** † MESA+ Institute for Nanotechnology, University of Twente, Enschede 7500 AE, The Netherlands; ‡ Institute of Physics, Czech Academy of Sciences, 162 00 Prague, Czech Republic; ¶ Optoelectronic Materials Section, Department of Chemical Engineering, Delft University of Technology, 2628 CN Delft, The Netherlands

## Abstract

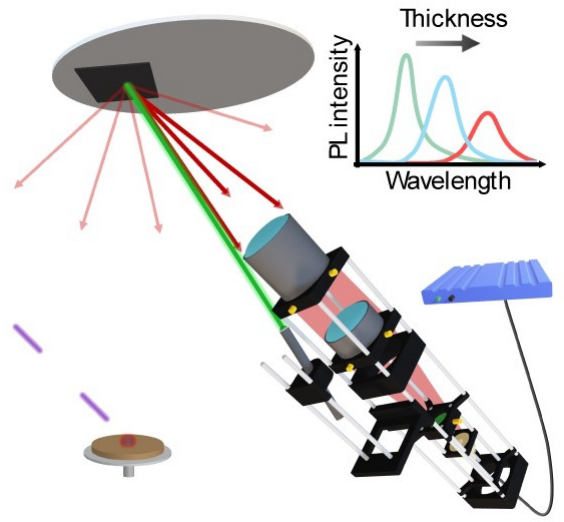

The potential of the vapor-phase deposition of metal halide perovskites (MHPs) for solar cells remains largely untapped, particularly in achieving rapid deposition rates. In this study, we employ in situ photoluminescence (PL) to monitor the growth dynamics of MHPs deposited via pulsed laser deposition (PLD), with rates ranging from 6 to 80 nm/min. Remarkably, the PL intensity evolution remains consistent across both low- and high-deposition rates, indicating that increased deposition rates do not significantly alter the fundamental mechanisms driving MHP formation via PLD. However, microstructural analysis and time-resolved microwave conductivity (TRMC) measurements reveal that increasing deposition rates lead to randomly oriented films on contact layers and reduced charge mobility compared with films grown at lower deposition rates. These findings emphasize the critical role of controlling initial nucleation and the value of in situ PL monitoring in optimizing the vapor-phase deposition of MHPs for enhanced photovoltaic performance at high deposition rates.

In the realm of renewable energy, the quest for efficient, cost-effective solar cell technologies has led to significant interest in metal halide perovskites (MHPs).^1,2^ These materials possess high absorption coefficients, tunable direct bandgaps, and long carrier diffusion lengths, resulting in high power conversion efficiencies (PCEs).^3^ The advancement of MHPs has primarily relied on solution-based methodologies, while physical vapor deposition (PVD) techniques have been less explored (with the exception of coevaporation). Nonetheless, PVD techniques are attractive to both the industrial and academic sectors, as these techniques could play an important role in the commercialization of MHPs in photovoltaics.^4,5^ Moreover, PVD techniques offer advantages such as conformal growth and precise control over film uniformity and thickness, critical factors for upscaling and incorporating thin films in heterostructures such as tandem solar cells. Despite these advantages, one of the main challenges facing vacuum deposition techniques is the typically low deposition rates, which need to be significantly increased to ensure high throughput in order to make these methods competitive for industrial use.^4^ The accessibility of wafer-scale pulsed laser deposition (PLD) systems brings PLD one step closer to scalability and improved throughput.^6−8^

PLD is a versatile thin film fabrication technique that employs laser energy to eject material from a single solid target in the form of a plasma plume that transfers the material onto a substrate where nucleation and growth processes occur.^9^ PLD’s primary strength lies in its capability to deposit complex chemical compositions, such as hybrid MHPs, from a single source. The latter typically consists of a customized ball-milled powder precursor mixture compacted into a target.^10−13^ Both PLD and the target fabrication are dry methods, meaning that the use of toxic solvents is avoided.^14^ Additionally, PLD allows for precise control over film thickness and deposition rates via varying the amount of laser pulses, laser energy, and laser pulse frequency, respectively.

Here, we demonstrate that increasing the laser pulse frequency from the previously optimized 4 Hz (6 nm/min) to 40 Hz (80 nm/min) results in a 13-fold increase in the deposition rate for MA_1–*x*_FA_*x*_PbI_3_ thin films. To further understand the growth of PLD-deposited perovskite films at various deposition rates, we integrated an in situ photoluminescence (PL) monitoring tool into the PLD chamber. These measurements offer valuable insights into defect formation processes, allowing us to directly compare the properties of MHP film growth at rates up to 13 times faster than our standard process. Additionally, we complemented our findings with microstructural analysis and time-resolved microwave conductivity (TRMC) measurements.^13,15^

## In Situ PL Monitoring during PLD of MHPs

**Figure 1:**
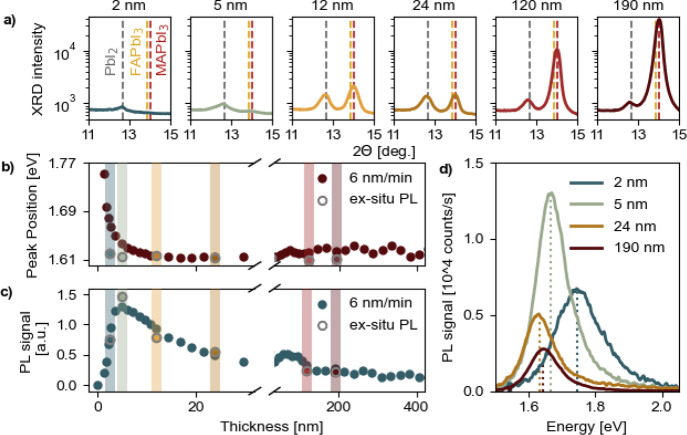
In situ PL evolution and complementary ex situ data of MA_1–*x*_FA_*x*_PbI_3_ samples grown on ITO/2PACz at a deposition rate of 6 nm/min. (a) Ex situ XRD patterns as a function of estimated thicknesses; the dashed lines in gray, yellow, and brown represent the PbI_2_ (100), FAPbI_3_ (100), and MAPbI_3_ (100) diffraction peaks, respectively. (b) PL peak position evolution during in situ measurements with scattered ex situ data points measured after deposition in ambient conditions for comparison. (c) PL signal evolution as a function of thickness with scattered normalized ex situ data points measured after deposition in ambient conditions. (d) Ex situ PL signal as a function of energy for thin films with varying thicknesses.

We showcase a comprehensive analysis of the PL intensity evolution during the deposition of MHPs on ITO/2PACz (2PACz: (2-[9*H*-carbazol-9-yl]ethyl) phosphonic acid) using PLD, initially at our optimized deposition rate of 6 nm/min. As a reference, we employed a well-established MA_1–*x*_FA_1–*x*_PbI_3_ recipe developed by our research team.^13,15^ Reference thin films of MA_0.55_FA_0.45_PbI_3_ were grown from a single source target (MAI:FAI:PbI_2_, 6:2:1; molar ratio) at a deposition rate of 6 nm/min and using a laser pulse frequency of 4 Hz. The evolution of the PL spectra was monitored during the entire deposition process by tracking the PL peak position and maximum intensity, as displayed in Figure 1b,c.

We observed a steep increase in the intensity of the PL signal during the initial stage of PLD, corresponding to the formation of MHP grain nuclei. The PL intensity reached an absolute maximum at 150 pulses (≈3.5 nm). Immediately after this point, a significant decrease in the PL intensity was recorded, which is attributed to the coalescence of grains and the formation of defective grain boundaries. This behavior is consistent with recent observations by Held et al. during the in situ PL monitoring of coevaporated MAPbI_3_ films at a deposition rate of 1.4 nm/min.^16^ The authors demonstrated that perovskite formation in the initial nucleation stage is almost defect-free, accompanied by high PL yields. However, once the perovskite grains begin to coalesce, it leads to compressive stress, forming defective grain boundaries and thus causing a significant PL quenching.^16,17^

The reduction in PL intensity is correlated with the formation of nonradiative recombination centers at these defective sites. Consequently, the PL signal remains almost constant, with a very slow decline and minor fluctuations until the end of the deposition. The oscillations in the PL signal/peak in the late stage of the deposition (>150 nm) are likely a consequence of the varying optical cavity effects.^18^ For the completed 400 nm thick MHP film, the PL intensity is typically about 10% of its maximum value for all samples, as shown in Figure 1d. This low PL intensity signal on thick films indicates the presence of nonradiative centers, which directly contribute to a decrease in quasi-Fermi level splitting, therefore leading to open circuit voltage losses at the solar cell device level, as observed in Figure S1 for the as-deposited thin films in a pin architecture.^19^

Moreover, the position of the PL peak during this initial stage of growth is markedly shifted to higher energies by 0.15 eV in comparison to that of the bulk (see Figure 1b,d). A compelling explanation for this phenomenon, supported by previous research, is the quantum confinement effects due to the formation of perovskite nanocrystals in the early stages of deposition.^16,20−24^ When these nanocrystals are excited by an external source, they emit radiation characterized by specific wavelengths that are intrinsically dependent on the nanocrystal’s size.^16,23,25,26^ Note that, during the initial stages of perovskite growth, accurately determining the thickness of the layer is challenging due to its nonhomogeneous nature. However, the thickness estimation presented here relied on the assumption of proportionality between the number of UV laser pulses and the thickness of the perovskite layer forming during PLD; see Figure S2. The specified thickness value at the beginning of the deposition is, therefore, only indicative.

To support these in situ measurements, we prepared a series of samples with approximate thicknesses of 2.4, 3.6, 12, 24, 120, and 190 nm to explore noteworthy regions using ex situ X-ray diffraction (XRD) and PL measurements in ambient conditions (22 °C, 30–40 RH); see Figure 1a and gray hollow points in panels b and c. The ex situ PL data (gray hollow points in Figure 1b) highlight the importance of in situ measurements as the peak position does not precisely align with the in situ PL data, particularly at the beginning of the deposition. At this stage, the PL peak position observed in ex situ measurements is significantly shifted to lower energies (bulk value). This shift could be caused by the dynamic nature of the PLD deposition process; removing the sample from the chamber may allow it to relax, potentially leading to surface rearrangements. Additionally, exposing the ultrathin film, composed of perovskite nanocrystals, to ambient conditions may also induce changes in the film’s properties. However, we do not attribute this shift to the act of breaking the vacuum itself. To test this, we measured the PL transition from the deposition pressure (0.02 mbar) to high vacuum (1·10^–7^ mbar) and observed no shift in the PL peak position. Figure 1a shows the evolution of the PbI_2_ (100) and perovskite (100) diffraction planes as the material grows along these preferential orientations. Notably, the first few nanometers (up to approximately 10 nm) of growth are PbI_2_-rich. This behavior has been observed previously for PLD and other vapor deposition methods, where the inorganic PbI_2_ tends to stick preferentially on the contact layer during the initial phase of deposition followed by reactions and interdiffusion with the organic components, ultimately leading to the formation of the MHP film.^13,27−30^

In addition, our data show the nontrivial nature of the PbI_2_ content in later deposition phases, suggesting an interplay between reactions and diffusion of the deposited and arriving material. While it may initially seem that only PbI_2_ is present during the early stages of growth, the strong PL signal suggests that perovskite has to also be present, as shown in Figure S3. This discrepancy is likely due to the limited signal-to-noise ratio of the XRD system used, which may not be sensitive enough to detect the perovskite phase at this stage. The PL signal of PbI_2_ can not be observed in the current experimental setup since the PL excitation energy (532 nm or 2.33 eV) is below the one of PbI_2_ (2.40 eV).

## In Situ PL Monitoring at Increased Deposition Rates

One of the most frequently mentioned requirements for the successful implementation of PVD methods for MHPs is the need to increase the deposition rate to meet industrial demands and achieve high throughput.^31^ To address this, we investigated the differences in optoelectronic and structural properties at various deposition rates by increasing the UV pulsed laser frequency from 4 Hz (6 nm/min) to 40 Hz (80 nm/min). This enhancement reduced the deposition time by a factor of 13, allowing a uniform coating of a 2.5 × 2.5 cm^2^ area for a 400 nm thick film in just 5 min (instead of 75 min); see Figure S4.

**Figure 2:**
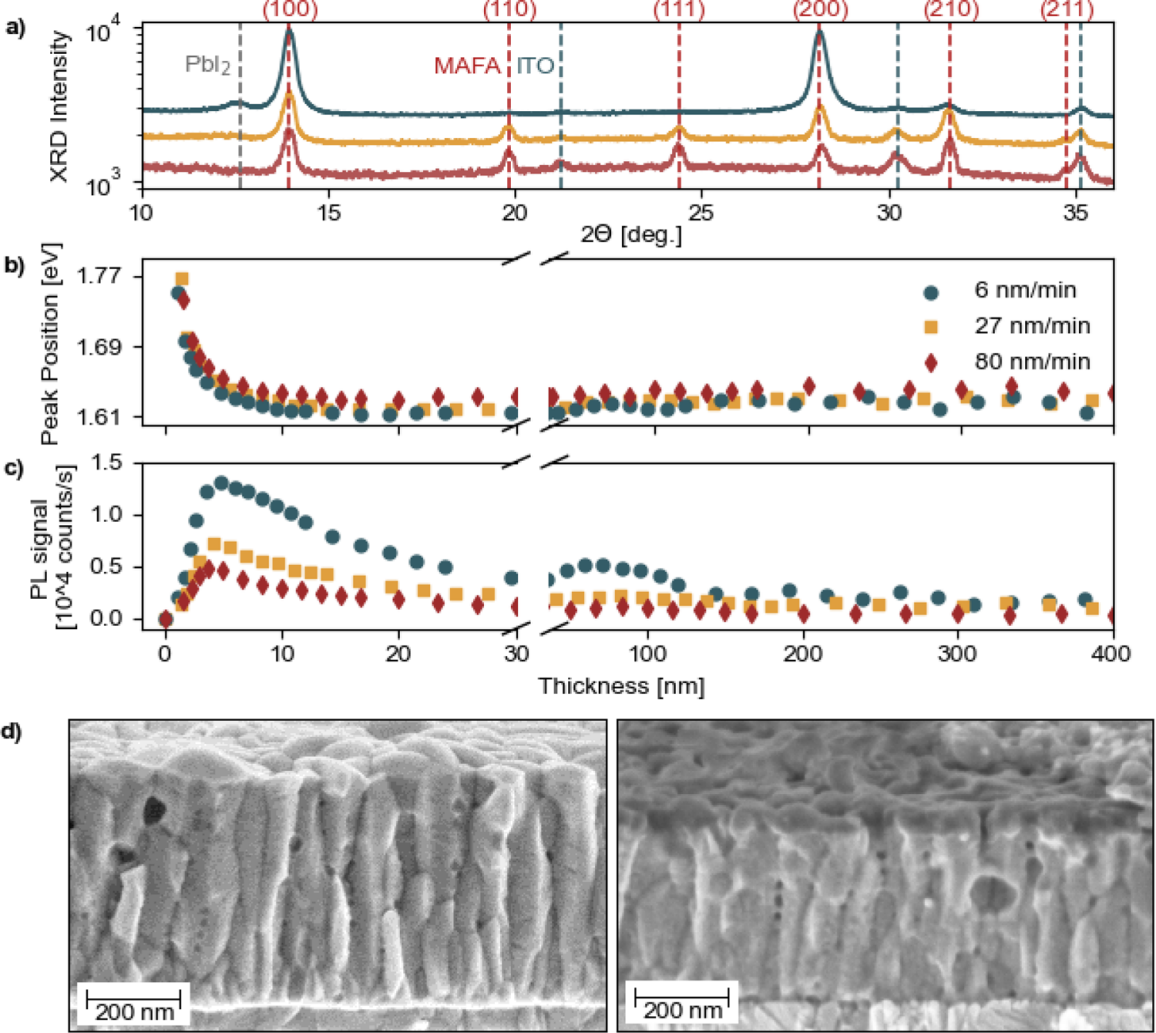
Structural, optical, and morphological comparison of MA_1–*x*_FA_*x*_PbI_3_ thin films grown on glass/ITO/2PACz substrates employing slow and accelerated deposition rates. (a) Ex situ XRD analysis of thin films deposited using different pulse laser frequencies in scanning mode; to enhance readability, the curves were artificially shifted along the *y*-axis. In situ PL evolution of (b) PL peak position and (c) PL signal for various deposition rates as a function of thickness. SEM cross-section image (d), absorber layer grown at 6 nm/min (left) in the ITO/2PACz/PVK stack and at 80 nm/min (right) in the ITO/2PACz/PVK/C60/BCP stack.

Figure 2b,c shows the evolution of the PL peak position and the PL signal at various deposition rates. Our observations indicate that higher deposition rates do not significantly affect the evolution of the PL signal. From this, we can conclude that the fundamental growth mechanisms of perovskite formation in the vapor phase during PLD remain unaffected despite variations in deposition rate. However, the overall absolute PL signal shows a slight decrease at higher deposition rates, suggesting an increased formation of defect states under these conditions. This is reflected in the lower photovoltaic performance of the cells at high deposition rates compared to that of the reference cells grown at low deposition rates (Figure S1).

The structural analysis of MA_1–*x*_FA_*x*_PbI_3_ films grown on ITO/2PACz using the scanning mode at deposition rates of 6 nm/min (400 nm), 27 nm/min (380 nm), and 80 nm/min (330 nm) is presented in Figure 2a. Note that the difference in thickness is due to the challenges imposed when employing PLD in the scanning mode, which will require a hardware modification for future experiments; see Figure S5. The film deposited at 6 nm/min shows distinct perovskite diffraction peaks at 2θ = 14.2° and 28.5°, corresponding to the cubic MA_1–*x*_FA_*x*_PbI_3_ (100) and (200) planes, indicating a strong preferential orientation along these planes. In contrast, the film grown at an 80 nm/min rate exhibits nearly anisotropic growth along all feasible diffraction planes. Similar XRD results were recently reported by Piot et al. during the fast growth of MAPbI_3_ via coevaporation.^32^ Additionally, the overall degree of crystallinity is lower for films grown at rapid deposition rates than for those grown at lower deposition rates. Furthermore, the suppression of the small PbI_2_ peak in the XRD pattern for films grown at rapid deposition rates^33,34^ suggests that the growth is more organic-rich at faster rates; the effect of modifying the target composition is presented in Figure S6. Thus, fine-tuning the target composition is most likely required when thin films are grown at rapid rates. Moreover, when comparing the medium deposition rate of 27 nm/min with the rapid 80 nm/min rate, we observed minimal differences in the XRD pattern. Given the complex relationship between crystal orientation and solar cell performance, understanding the full impact on overall performance remains challenging and requires a further dedicated study.^35,36^

Figure 2d displays the scanning electron microscopy (SEM) cross-section image revealing a columnar grain growth structure for films deposited at both low and high deposition rates, which is advantageous for vertical charge transport on finalized solar cells. Larger grain sizes are still preferable, as fewer recombination centers at grain boundaries help facilitate more efficient charge extraction.^37^ AFM images in Figure S7a,b show similar surface morphologies for both films grown at slow and rapid deposition rates, with a root-mean-square roughness of *r*_RSM_ = 21 nm measured over a 5 × 5 μm area. A more detailed analysis reveals that the AFM image for a slower deposition rate (Figure S7a) shows approximately 24grains/μm^2^ with an average grain size of about 110 nm. In contrast, the AFM image for a faster deposition rate (Figure S7b) contains approximately 43grains/μm^2^ with highly inconsistent grain sizes ranging from 40 to 250 nm. This variation may result in local inhomogeneities that affect the electrical properties and, generally, a less compact thin film, which is undesirable for PSCs.^38^

## In Situ PL Monitoring of MA_1–*x*_FA_*x*_PbI_3_ with and without PbCl_2_ at High Deposition Rates and Electronic Characterization

**Figure 3:**
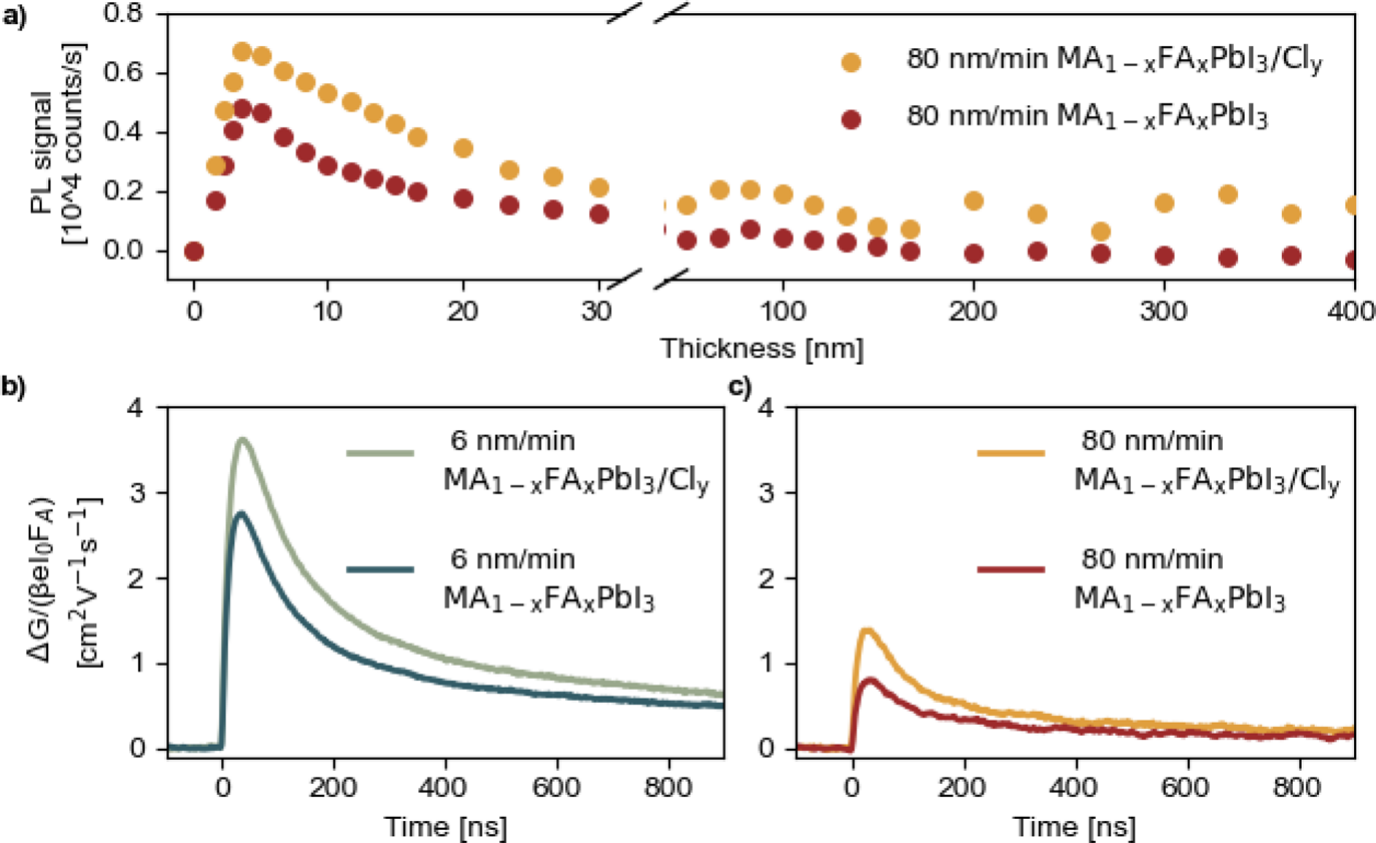
(a) In situ photoluminescence (PL) analysis during the PLD growth of MA_1–*x*_FA_*x*_PbI_3_ and MA_1–*x*_FA_*x*_PbI_3_/Cl_*y*_ at 80 nm/min deposition rate. Photoconductance traces for PLD grown MA_1–*x*_FA_*x*_PbI_3_ and MA_1–*x*_FA_*x*_PbI_3_/Cl_*y*_ films with 6 nm/min (b) and 80 nm/min (c) deposition rates.

Leveraging the in situ PL capabilities, we conducted further in situ PL measurements during the PLD growth of MA_1–*x*_FA_*x*_PbI_3_ on glass/ITO/2PACz substrates with a modified composition by partially substituting PbI_2_ with 20 mol % of PbCl_2_ in the target mixture.^27,39^ Previously, we observed improvements in morphology, crystallinity, and performance with this substitution, specifically in the fill factor (FF) of solar cells, leading to a 1% gain in power conversion efficiency despite the no detectable presence of PbCl_2_ in the final composition.^27^ The addition of chloride-based additives has been widely reported to slow down the crystallization dynamics, act as a bulk defect passivation strategy, enhance charge carrier lifetimes, and improve the overall performance of perovskite solar cells (PSCs).^27,39−41^ As summarized in Figure 3a, the PL evolution remained virtually unchanged for both composition variants and deposition rates, indicating a broadly applicable growth mechanism of perovskite film formation via PLD. Yet, a slight delay in the PL decay/coalescence of the individual grains indicates a reduced nucleation density when PbCl_2_ is added to the target mixture. This results in lower overall defect densities in the films and therefore in a higher final PL signal value as compared to the film without PbCl_2_.

Additionally, the XRD patterns in Figure S8a show that the addition of PbCl_2_ not only preserves the preferential orientation growth along the [001] direction but also enhances the overall crystallinity of the films even at higher deposition rates. This is also visible in the SEM top-view images in Figures S8b–e. In detail, the SEM images show that the MA_1–*x*_FA_*x*_PbI_3_/Cl_*y*_ film deposited at a high rate presents grains with textured surfaces and more defined boundaries compared to the corresponding SEM image without PbCl_2_. These findings confirm the benefits of incorporating PbCl_2_ to enhance the crystallization process and diminish the nonradiative defect density.

Time-resolved microwave conductivity (TMRC) measurements were performed to study charge carrier dynamics of chloride and nonchloride passivated PLD grown films, respectively, MA_1–*x*_FA_*x*_PbI_3_/Cl_*y*_ and MA_1–*x*_FA_*x*_PbI_3_, at different deposition rates. Note that samples employed for this experiment were deposited on fused silica, and changes in the crystallization dynamics due to the different surface polarity between 2PACz and fused silica are expected; see Figure S8. The corresponding TRMC signals are shown in Figure 3b,c. Generally, the initial increase in the signal originates from the generation of free charge carriers by a short laser pulse. The decay of the signal is attributed to the immobilization of excess charge carriers via trapping or the recombination of electrons and holes. Thus, a less pronounced decay is related to fewer recombination processes.

In detail, the maximum TRMC signal is related to the product between the charge carrier photoconversion yield and mobility sum. The maximum TRMC signal corresponds to the mobility sum when considering the photoconversion yield equal to 1 for these perovskites at room temperature. Therefore, the mobility sum is ∼3 cm^2^ V^–1^ s^–1^ for a low deposition rate and ∼1 cm^2^ V^–1^ s^–1^ for a high deposition rate, which are comparable to values reported previously for perovskite thin films deposited by vacuum-based techniques.^42^ The relatively low TRMC values are related to the small grains limiting the mobility, thus the potential device fill factor.^43^Figure 3c displays the noticeably reduced TRMC signals for the case of accelerated deposition rates, likely as a result of increased defect densities due to poorer structural properties (crystallinity, density). Nevertheless, we observed a small (≈20%) but reproducible increase in the maximum TRMC signal upon addition of PbCl_2_, independent of the deposition rate. Moreover, as shown for the intensity-normalized TRMC traces recorded at different intensities in Figure S9, a more pronounced second-order recombination is visible upon PbCl_2_ addition. Indeed, when the dynamics is dominated by second-order recombination, the TRMC signal becomes lower with increasing laser intensity due to the corresponding increasing concentration of photogenerated carriers band–band recombination. We attribute the rise in the TRMC signal and the enhanced second-order behavior to the passivation of crystal defects, as previously discussed.^39,44^ These findings further confirm the positive impact of PbCl_2_ addition, even at high deposition rates, now verified by charge carrier dynamics measurements.

In summary, we employed PLD as a PVD technique for the growth of MHPs at elevated deposition rates ranging from 6 to 80 nm/min. The latter was realized by changing the laser pulse frequency from 4 to 40 Hz. By in situ monitoring the growth evolution of MA_1–*x*_FA_*x*_PbI_3_/Cl_*y*_ films during PLD, we found that the fundamental growth mechanisms remain consistent across this range of deposition rates. However, the crystallinity, photovoltaic performance, and mobility of the rapidly deposited thin films are lower than those of the reference films deposited at the standard 4 Hz rate. Furthermore, the incorporation of bulk passivation using PbCl_2_ has shown improvements in overall film quality. This study also highlights the importance of in situ monitoring of perovskite growth and the exceptional versatility of PLD, which allows rapid adjustment of deposition conditions, such as switching targets or interrupting deposition within seconds. This advancement offers an opportunity to achieve better control over crucial solar cell parameters, such as open-circuit voltage, through PLQY evaluations and fill factor by charge carrier mobility measurements. This presents significant potential for exploring novel photovoltaic materials and a variety of PVD passivation techniques that still need to be unveiled.

## Experimental Methods

### Target Preparation

All the chemicals were purchased in powder form and used without further purification: methylammonium iodide (MAI, >99.99% greatcell solar), formamidinium iodide (FAI, >99.99% greatcell solar), lead iodide (PbI_2_, 99.999% Sigma-Aldrich), and lead chloride (PbCl_2_, ultradry, 99.999%, Alfa Aesar). Powders were mixed inside a N_2_ glovebox in nonstoichiometric ratios as previously optimized, 1:8 inoganic:organic precursor ratio and MAI:FAI 75:25 molar ratio.^13,15,27^ Subsequently, the precursors were ball-milled and pressed using 470 MPa to form a ≈2.5 mm thick disc of 20 mm diameter.

### Pulsed Laser Deposition (PLD)

Samples were grown in a customized (TSST Demcon) vacuum chamber (base pressure ≈1 × 10^–7^ mbar) in an Ar atmosphere (working pressure: 0.02 mbar). The material is ablated from a solid rotating target to achieve uniform ablation by employing a Coherent KrF excimer laser (λ = 248 nm). The laser fluence is then adjusted to 0.31 J·cm^–2^ for all depositions. The spot size is set by the metal mask to 2.33 mm^2^. A target-to-substrate distance was kept constant at 55 mm. To ensure homogeneous coverage, the sample stage with substrates was scanned in a square scanning pattern of 36 × 36 mm^2^ at varying speeds from 1.5 to 2 mm/s. The pulse laser frequency was varied in the range from 4 to 40 Hz (≈6 to 80 nm/min) to achieve rapid deposition rates. The number of laser pulses was modified to complete full scanning cycles for the experiments requiring higher pulsed laser frequencies. The stationary mode was employed during in situ PL measurements.

### X-ray Diffraction (XRD)

XRD patterns were measured using PANalytical X’Pert PRO with a Cu X-ray source in an ambient atmosphere at room temperature.

### Photoluminescence Spectroscopy (PL)

Ex situ PL spectra were measured by using a custom-built setup. The 520 nm fiber-coupled laser diode module with adjustable current (Matchbox series) was used as an excitation source.

In situ PL spectra were measured with a long-distance self-built PL setup designed by using the Thorlabs cage system and Thorlabs optical components. As an excitation source, a focused 532 nm collimated laser-diode-pumped DPSS Laser Module with a constant power of 4.5 mW (round beam) was employed. The distance between the sample and the objective lens was 301 mm. A fiber-coupled StellarNet BLUE-Wave Miniature Spectrometer was used in both PL setups. A thickness correction for the PL intensity has been applied as shown in Figure S2.

### Device Preparation

ITO glass substrates (20 × 15 mm, Ossila) were cleaned in ultrasonic baths sequentially containing a 2% v/v solution of Hellmanex III, acetone, 2-propanol, and deionized water, each for 10 min, followed by a 15 min UV-Ozone treatment. Immediately after this, the substrates were loaded into a N_2_ glovebox to statically coat self-assemble monolayers via spin-coating (100 μL, 5000 rpm for 30 s, 2PACz, TCI America, 1 mg/mL in Ethanol, stirred overnight). After this, the ITO/2PACz substrates were annealed at 100 °C for 10 min, followed by a washing step (2 × 100 μL of ethanol dynamically added (5000 rpm for 30 s)). The MHP absorber was deposited using PLD. After deposition, the ITO/2PACz/PLD-Perovskite samples were coated with 25 nm of C_60_ (99.5%, Ossila), 7 nm of BCP (Lumtec), and 100 nm of Ag (99.99%, Kurt J. Lesker) via thermal evaporation (NANOVAK evaporator). The contact layer thickness was monitored with a quartz crystal microbalance (QCM).

### Device Characterization

The *J*–*V* characteristics of the devices were recorded using a solar simulator (SINUS-70 ADVANCED, Wavelabs) and an Ossila Source-Measure Unit under AM1.5G spectrum illumination (ambient conditions 30–35% humidity) in both forward (Jsc → Voc) and reverse (Voc → Jsc) directions with a scan rate of 0.11 V s^–1^ and illuminated masked area of 1 mm^2^. The intensity was calibrated with a monocrystalline silicon WPV N-type broadband reference solar cell to match the global standard AM 1.5 G spectrum with an intensity of 100 mW cm^–2^.

### Time-Resolved Microwave Conductivity (TRMC)

During TRMC measurements, an ultrafast Nd:YAG (λ = 650 nm) laser with pulses of the duration of ≈3.5 ns on average at a repetition of 10 Hz is used to photogenerate charge carriers in the perovskite layer. The light intensity of the excitation laser pulse is tuned between 10^9^ and 10^12^ photons cm^–2^ by using an array of neutral density filters. At the same time, microwaves generated by a voltage-controlled oscillator with frequencies between 8.2 and 12.2 GHz pass through the perovskite sample located in a microwave cavity cell. The cavity enables highly sensitive measurements by creating a standing wave at a specific resonant microwave frequency whose maximum electrical field overlaps with the sample. The reduction of the microwave power as a function of the time elapsed after the laser pulse Δ*P*(*t*) resulting from the interaction between microwaves and the photogenerated free (mobile) carriers is then recorded. The intensity-normalized reduction in microwave power can be related to the time-dependent variation in the conductance Δ*G*(*t*) of the perovskite thin film, which is related to the integrated change in the electrical conductivity Δσ(*t*) over the full perovskite layer thickness. This scales with the time-dependent concentrations of free electrons and holes and their mobilities sum (Σσ = σ_e_ + σ_h_). Hence, the maximum TRMC signal, expressed by Δ*G*_max_ normalized by the intensity of the laser *I*_0_, the form factor β, and the elementary charge *e* and corrected for the absorbed fraction of light *F*_A_ at the excitation wavelength, can be expressed by the product between the charge carriers photoconversion yield ϕ and Σσ. If every absorbed photon generates a single electron–hole pair, which commonly occurs in direct bandgap perovskites with low exciton binding energy at room temperature, ϕ is equal to 1. Hence, the maximum TRMC signal enables one to estimate Σσ.

## Supporting Information Available

*J*–*V* curve statistics, PL signal thickness correction function, ex situ PL data, pulse–thickness dependence, visualization of PLD substrate scanning limitation, PL composition dependence, AFM images, XRD data, SEM images, TRMC data, and AFM images during nucleation phase (PDF)

## References

[ref1] Jean J.; Brown P. R. Emerging Photovoltaic Technologies; IOP Publishing, 2020.

[ref2] Culik P.; Brooks K.; Momblona C.; Adams M.; Kinge S.; Maréchal F.; Dyson P. J.; Nazeeruddin M. K. Design and cost analysis of 100 MW perovskite solar panel manufacturing process in different locations. ACS Energy Lett. 2022, 7 (9), 3039–3044. 10.1021/acsenergylett.2c01728.

[ref3] National Renewable Energy Laboratory (NREL) Best Research-Cell Efficiency Chart. https://www.nrel.gov/pv/cell-efficiency.html (accessed 2025-01–17).

[ref4] Abzieher T.; et al. Vapor Phase Deposition of Perovskite Photovoltaics: Short Track to Commercialization?. Energy Environ. Sci. 2024, 17 (5), 1645–1663. 10.1039/D3EE03273F.

[ref5] Soto-Montero T.; Soltanpoor W.; Morales-Masis M. Pressing challenges of halide perovskite thin film growth. APL materials 2020, 8 (11), 110903 10.1063/5.0027573.

[ref6] Smirnov Y.; Repecaud P.-A.; Tutsch L.; Florea I.; Zanoni K. P.; Paliwal A.; Bolink H. J.; i Cabarrocas P. R.; Bivour M.; Morales-Masis M. Wafer-scale pulsed laser deposition of ITO for solar cells: reduced damage vs. interfacial resistance. Materials Advances 2022, 3 (8), 3469–3478. 10.1039/D1MA01225H.

[ref7] Soltanpoor W.; Bracesco A. E.; Rodkey N.; Creatore M.; Morales-Masis M. Low damage scalable pulsed laser deposition of sno2 for p–i–n perovskite solar cells. Solar RRL 2023, 7 (23), 2300616 10.1002/solr.202300616.

[ref8] Zanoni K. P.; Paliwal A.; Hernández-Fenollosa M. A.; Repecaud P.-A.; Morales-Masis M.; Bolink H. J. ITO Top-Electrodes via Industrial-Scale PLD for Efficient Buffer-Layer-Free Semitransparent Perovskite Solar Cells. Advanced Materials Technologies 2022, 7 (10), 2101747 10.1002/admt.202101747.

[ref9] Eason R. Pulsed Laser Deposition of Thin Films: Applications-Led Growth of Functional Materials; Wiley, 2007.

[ref10] Prochowicz D.; Saski M.; Yadav P.; Gratzel M.; Lewinski J. Mechanoperovskites for photovoltaic applications: preparation, characterization, and device fabrication. Acc. Chem. Res. 2019, 52 (11), 3233–3243. 10.1021/acs.accounts.9b00454. 31702124

[ref11] Rosales B. A.; Wei L.; Vela J. Synthesis and mixing of complex halide perovskites by solvent-free solid-state methods. Journal of solid state chemistry 2019, 271, 206–215. 10.1016/j.jssc.2018.12.054.

[ref12] Shepelin N. A.; Tehrani Z. P.; Ohannessian N.; Schneider C. W.; Pergolesi D.; Lippert T. A practical guide to pulsed laser deposition. Chem. Soc. Rev. 2023, 52 (7), 2294–2321. 10.1039/D2CS00938B. 36916771 PMC10068590

[ref13] Soto-Montero T.; Kralj S.; Soltanpoor W.; Solomon J. S.; Gómez J. S.; Zanoni K. P.; Paliwal A.; Bolink H. J.; Baeumer C.; Kentgens A. P.; Morales-Masis M. Single-Source Vapor-Deposition of MA_*1–x*_FA_*x*_PbI_3_ Perovskite Absorbers for Solar Cells. Adv. Funct. Mater. 2024, 34 (50), 2300588 10.1002/adfm.202300588.

[ref14] Ávila J.; Momblona C.; Boix P. P.; Sessolo M.; Bolink H. J. Vapor-deposited perovskites: the route to high-performance solar cell production?. Joule 2017, 1 (3), 431–442. 10.1016/j.joule.2017.07.014.

[ref15] Soto-Montero T.; Soltanpoor W.; Kralj S.; Birkhölzer Y. A.; Remes Z.; Ledinsky M.; Rijnders G.; Morales-Masis M. Single-source pulsed laser deposition of MAPbI_3_. 2021 IEEE 48th Photovoltaic Specialists Conference (PVSC); 2021; pp 1318–1323.

[ref16] Held V.; Mrkyvkova N.; Nadazdy P.; Vegso K.; Vlk A.; Ledinsky M.; Jergel M.; Chumakov A.; Roth S. V.; Schreiber F.; Siffalovic P. Evolution of Structure and Optoelectronic Properties During Halide Perovskite Vapor Deposition. J. Phys. Chem. Lett. 2022, 13 (51), 11905–11912. 10.1021/acs.jpclett.2c03422. 36525260

[ref17] Held V.; Mrkyvkova N.; Halahovets Y.; Nadazdy P.; Vegso K.; Vlk A.; Ledinsky M.; Jergel M.; Bernstorff S.; Keckes J.; Schreiber F.; Siffalovic P. Evolution of Defects, Morphology, and Strain during FAMAPbI3 Perovskite Vacuum Deposition: Insights from In Situ Photoluminescence and X-ray Scattering. ACS Appl. Mater. Interfaces 2024, 16 (27), 35723–35731. 10.1021/acsami.4c04095. 38935890

[ref18] Patel J. B.; Wright A. D.; Lohmann K. B.; Peng K.; Xia C. Q.; Ball J. M.; Noel N. K.; Crothers T. W.; Wong-Leung J.; Snaith H. J. others Light absorption and recycling in hybrid metal halide perovskite photovoltaic devices. Adv. Energy Mater. 2020, 10 (10), 1903653 10.1002/aenm.201903653.

[ref19] Caprioglio P.; Stolterfoht M.; Wolff C. M.; Unold T.; Rech B.; Albrecht S.; Neher D. On the relation between the open-circuit voltage and quasi-fermi level splitting in efficient perovskite solar cells. Adv. Energy Mater. 2019, 9 (33), 1901631 10.1002/aenm.201901631.

[ref20] Mrkyvkova N.; Held V.; Nadazdy P.; Subair R.; Majkova E.; Jergel M.; Vlk A.; Ledinsky M.; Kotlar M.; Tian J.; Siffalovic P. Combined in situ photoluminescence and x-ray scattering reveals defect formation in lead-halide perovskite films. J. Phys. Chem. Lett. 2021, 12 (41), 10156–10162. 10.1021/acs.jpclett.1c02869. 34637618

[ref21] Wagner L.; Mundt L. E.; Mathiazhagan G.; Mundus M.; Schubert M. C.; Mastroianni S.; Würfel U.; Hinsch A.; Glunz S. W. Distinguishing crystallization stages and their influence on quantum efficiency during perovskite solar cell formation in real-time. Sci. Rep. 2017, 7 (1), 14899 10.1038/s41598-017-13855-6. 29097712 PMC5668251

[ref22] White L. R.; Kosasih F. U.; Ma K.; Fu J.; Feng M.; Sherburne M. P.; Asta M.; Sum T. C.; Mhaisalkar S. G.; Bruno A. MAPbI3 Perovskite Multiple Quantum Wells for Enhanced Light Emission and Detection. ACS Energy Lett. 2024, 9 (9), 4450–4458. 10.1021/acsenergylett.4c01499.

[ref23] Malgras V.; Tominaka S.; Ryan J. W.; Henzie J.; Takei T.; Ohara K.; Yamauchi Y. Observation of quantum confinement in monodisperse methylammonium lead halide perovskite nanocrystals embedded in mesoporous silica. J. Am. Chem. Soc. 2016, 138 (42), 13874–13881. 10.1021/jacs.6b05608. 27667498

[ref24] Parrott E. S.; Patel J. B.; Haghighirad A.-A.; Snaith H. J.; Johnston M. B.; Herz L. M. Growth modes and quantum confinement in ultrathin vapour-deposited MAPbI 3 films. Nanoscale 2019, 11 (30), 14276–14284. 10.1039/C9NR04104D. 31317998

[ref25] Anaya M.; Rubino A.; Rojas T. C.; Galisteo-López J. F.; Calvo M. E.; Míguez H. Strong quantum confinement and fast photoemission activation in CH3NH3PbI3 perovskite nanocrystals grown within periodically mesostructured films. Advanced Optical Materials 2017, 5 (8), 1601087 10.1002/adom.201770043.

[ref26] Zhang F.; Huang S.; Wang P.; Chen X.; Zhao S.; Dong Y.; Zhong H. Colloidal synthesis of air-stable CH3NH3PbI3 quantum dots by gaining chemical insight into the solvent effects. Chem. Mater. 2017, 29 (8), 3793–3799. 10.1021/acs.chemmater.7b01100.

[ref27] Soto-Montero T.; et al. Single-source pulsed laser-deposited perovskite solar cells with enhanced performance via bulk and 2D passivation. Joule 2024, 8 (12), 3412–3425. 10.1016/j.joule.2024.09.001.

[ref28] Abzieher T.; Feeney T.; Schackmar F.; Donie Y. J.; Hossain I. M.; Schwenzer J. A.; Hellmann T.; Mayer T.; Powalla M.; Paetzold U. W. From Groundwork to Efficient Solar Cells: On the Importance of the Substrate Material in Co-Evaporated Perovskite Solar Cells. Adv. Funct. Mater. 2021, 31 (42), 2104482 10.1002/adfm.202104482.

[ref29] Guesnay Q.; Sahli F.; Ballif C.; Jeangros Q. Vapor deposition of metal halide perovskite thin films: Process control strategies to shape layer properties. Apl Materials 2021, 9 (10), 100703 10.1063/5.0060642.

[ref30] Kim B.-S.; Gil-Escrig L.; Sessolo M.; Bolink H. J. Deposition kinetics and compositional control of vacuum-processed CH3NH3PbI3 perovskite. J. Phys. Chem. Lett. 2020, 11 (16), 6852–6859. 10.1021/acs.jpclett.0c01995. 32701293

[ref31] Wassweiler E.; Panda A.; Kadosh T.; Nguyen T.; Hsu W.-J.; Pettit E.; Holmes R. J.; Tuller H.; Bulovic V. Design of a custom vapor transport co-deposition system for scalable production of perovskite solar cells. Journal of Vacuum Science & Technology A 2023, 41 (5), 052801 10.1116/6.0002668.

[ref32] Piot M.; Alonso J. E. S.; Zanoni K. P.; Rodkey N.; Ventosinos F.; Roldán-Carmona C.; Sessolo M.; Bolink H. Fast Coevaporation of 1 μm Thick Perovskite Solar Cells. ACS Energy Lett. 2023, 8 (11), 4711–4713. 10.1021/acsenergylett.3c01724. 37969254 PMC10644377

[ref33] Jacobsson T.; Correa-Baena J.-P.; halvani anaraki E.; Philippe B.; Stranks S.; Bouduban M.; Tress W.; Schenk K.; Teuscher J.; Moser J.-E.; Rensmo H.; Hagfeldt A. Unreacted PbI2 as a double-edged sword for enhancing the performance of perovskite solar cells. J. Am. Chem. Soc. 2016, 138 (32), 10331–10343. 10.1021/jacs.6b06320. 27437906

[ref34] Park B.-w.; Kedem N.; Kulbak M.; Lee D.; Yang W.; Jeon N.; Seo J.; Kim G.; Kim K.; Shin T.; Hodes G.; Cahen D.; Seok S. I. Understanding how excess lead iodide precursor improves halide perovskite solar cell performance. Nat. Commun. 2018, 9 (1), 3301 10.1038/s41467-018-05583-w. 30120225 PMC6098034

[ref35] Li B.; Shen T.; Yun S. Recent progress of crystal orientation engineering in halide perovskite photovoltaics. Materials Horizons 2023, 10 (1), 13–40. 10.1039/D2MH00980C. 36415914

[ref36] Fang Z.; Yan N.; Liu S. Modulating preferred crystal orientation for efficient and stable perovskite solar cells—From progress to perspectives. InfoMat 2022, 4 (10), e12369 10.1002/inf2.12369.

[ref37] Dong H.; Ran C.; Gao W.; Li M.; Xia Y.; Huang W. Metal Halide Perovskite for next-generation optoelectronics: progresses and prospects. ELight 2023, 3 (1), 3 10.1186/s43593-022-00033-z.

[ref38] An Q.; Paulus F.; Becker-Koch D.; Cho C.; Sun Q.; Weu A.; Bitton S.; Tessler N.; Vaynzof Y. Small grains as recombination hot spots in perovskite solar cells. Matter 2021, 4 (5), 1683–1701. 10.1016/j.matt.2021.02.020.

[ref39] Lohmann K.; Motti S.; Oliver R.; Ramadan A.; Sansom H.; Yuan Q.; Elmestekawy K.; Patel J.; Ball J.; Herz L.; Snaith H.; Johnston M. Solvent-free method for defect reduction and improved performance of pin vapor-deposited perovskite solar cells. ACS Energy Lett. 2022, 7 (6), 1903–1911. 10.1021/acsenergylett.2c00865. 35719271 PMC9199003

[ref40] Babaei A.; Soltanpoor W.; Tesa-Serrate M. A.; Yerci S.; Sessolo M.; Bolink H. J. Preparation and Characterization of Mixed Halide MAPbI3- xClx Perovskite Thin Films by Three-Source Vacuum Deposition. Energy Technology 2020, 8 (4), 1900784 10.1002/ente.201900784.

[ref41] Stranks S. D.; Eperon G. E.; Grancini G.; Menelaou C.; Alcocer M. J.; Leijtens T.; Herz L. M.; Petrozza A.; Snaith H. J. Electron-hole diffusion lengths exceeding 1 micrometer in an organometal trihalide perovskite absorber. Science 2013, 342 (6156), 341–344. 10.1126/science.1243982. 24136964

[ref42] Momblona C.; Gil-Escrig L.; Bandiello E.; Hutter E. M.; Sessolo M.; Lederer K.; Blochwitz-Nimoth J.; Bolink H. J. Efficient vacuum deposited pin and nip perovskite solar cells employing doped charge transport layers. Energy Environ. Sci. 2016, 9 (11), 3456–3463. 10.1039/C6EE02100J.

[ref43] Vaynzof Y. The future of perovskite photovoltaics—thermal evaporation or solution processing?. Adv. Energy Mater. 2020, 10 (48), 2003073 10.1002/aenm.202003073.

[ref44] Li H.; Zhou J.; Tan L.; Li M.; Jiang C.; Wang S.; Zhao X.; Liu Y.; Zhang Y.; Ye Y.; Tress W.; Yi C. Sequential vacuum-evaporated perovskite solar cells with more than 24% efficiency. Science Advances 2022, 8 (28), eabo7422 10.1126/sciadv.abo7422. 35857518 PMC10942770

