# Supplementary material for: Pulsed Laser Deposition of Halide Perovskites with over 10-Fold Enhanced Deposition Rates
Source: J Phys Chem Lett. 2025 Jan 31;16(6):1453–60. doi: 10.1021/acs.jpclett.5c00047 (PMC11831724; doi:10.1021/acs.jpclett.5c00047)
Supplement: Supplementary file 1 — jz5c00047_si_002.pdf [file jz5c00047_si_002.pdf]

Supporting Information

Pulsed Laser Deposition of Halide Perovskites with over 10-fold Enhanced Deposition Rates

Vojta Kliner,[*,†,‡] Tatiana Soto-Montero,[†] Jasmeen Nespoli,[¶] Tom J. Savenije,[¶] Martin Ledinský,[‡] and Monica Morales-Masis[*,†]

†*MESA+ Institute for Nanotechnology, University of Twente, Enschede 7500 AE, The Netherlands*

‡*Institute of Physics, Czech Academy of Sciences, 162 00 Prague, Czech Republic*

¶*Optoelectronic Materials Section, Department of Chemical Engineering, Delft University of Technology, 2628 CN Delft, The Netherlands*

E-mail: kliner@fzu.cz; m.moralesmasis@utwente.nl

Supporting Information Available

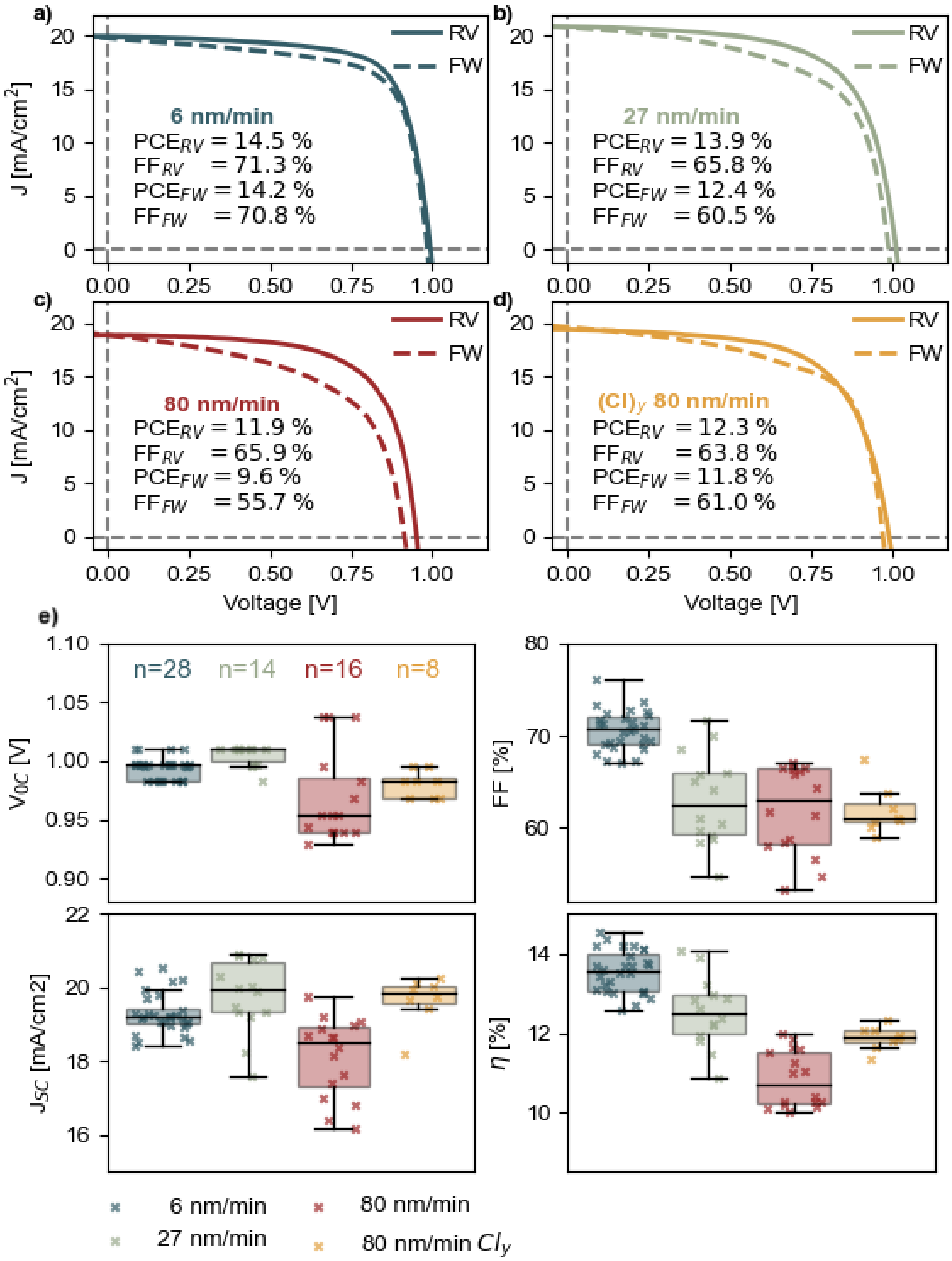

Figure S1: Current-voltage (a-d.) measurements of best performing photovoltaic devices ITO/2PACz/PLD-perovskite(as deposited)/C60/BCP/Ag under AM1.5 illumination, as measured under reverse bias (solid line) and forward bias (dashed line). The J-V statistic of working cells d.

Proof-of-concept solar cells were made without previous optimization (with the exception of a.), thus serving only as a demonstration of working devices. In Figure S1.a-c. J–V curves of devices made out of $MA_{1-x}A_xPbI$ (absorber layer) for different deposition rates can be seen. In the last case, Figure S1.d, the $MA_{1-x}FA_xPbI_3/Cl_y$ was used. In the figure S1.e, the J-V statistic of working cells is provided.

At the initial stages of deposition, the film thickness is insufficient to absorb all the excitation light. To address this, a thickness correction function, y, has been applied to the PL intensity in Figure S2. Thus, the shown PL signal is proportional to the photoluminescence quantum yield (PLQY). A series of samples with different thicknesses were deposited. Then, the transmissivity was measured, and the dataset was fitted with Beer-Lamberts law at 532 nm a wavelength to obtain a correction function y.

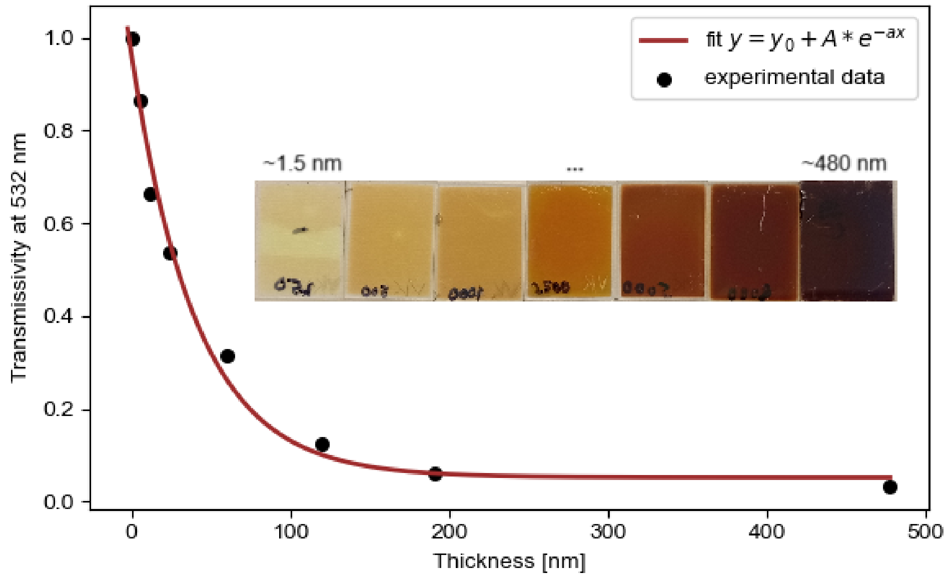

Figure S2: Dataset and fit employed to make the thickness correction. In our case, we used the values A=0.913, y0=0.052, and t=1712.4.

Figure S3 shows the ex-situ PL measurements (conducted in air) for ultrathin samples, indicating the presence of perovskite as the PL peak position corresponds to the characteristic perovskite emission. This confirms that perovskite is present even in these ultrathin samples despite being undetectable by XRD. The lack of the XRD signal is likely caused due to the

limited XRD signal-to-noise ratio. The XRD system used has detection limits in the range of 0.1 to 1 wt.% per phase, making it suitable for quality control applications (as noted in "Phase Quantification Using XRD | Malvern Panalytical") but likely too insensitive for detecting such small amounts of material in these ultrathin films.

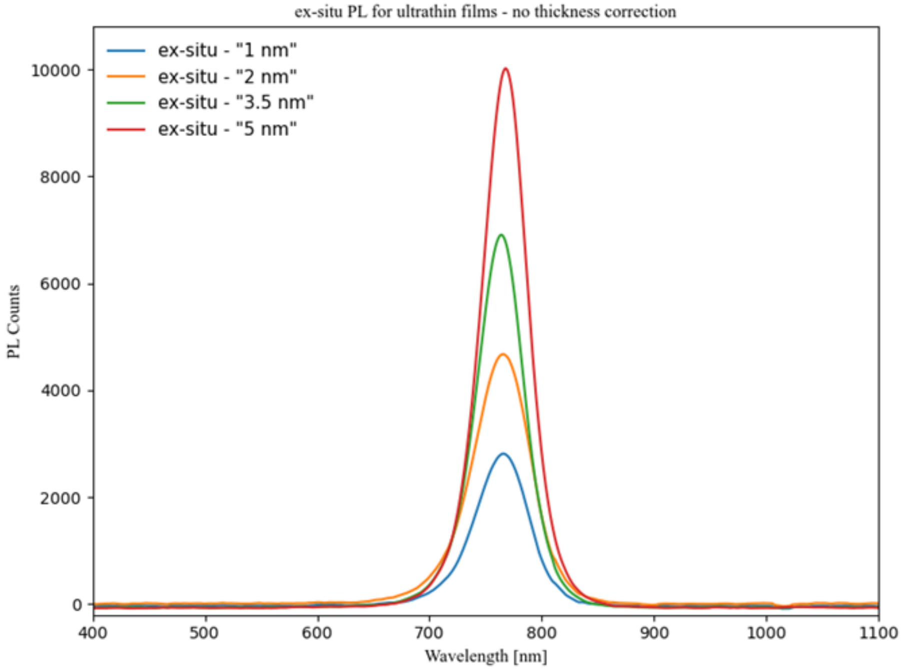

Figure S3: Ex-situ PL (in air) for ultrathin MHP samples.

In Figure S4.a the estimated linear correlation between the film thickness and the number of UV laser pulses shot to a single source target during PLD deposition at various deposition frequencies can be seen. It is based on SEM and profilometer measurements of final films. The estimation is not fulfilled during the initial stages of perovskite growth (first $\sim$ 10 nm) due to its non-homogeneous nature. The specified thickness value at the beginning of deposition is only indicative.

Please note that the behavior observed in Figure S4.b, where less pulses (and thus less evaporated material) is required to achieve a certain film thickness at higher deposition rates, is unexpected. This complex phenomenon is not yet fully understood. Our current hypothesis suggests that an "atmosphere" composed of organic compounds—arising from

the fast evaporation of the organic-rich target forms within the deposition chamber. This atmosphere then facilitates increased particle sticking on the substrate at higher deposition rates since the vacuum pump and argon flow are insufficient to keep the chamber clean at these high deposition rates, so we are utilizing even some of the organic particles that would normally miss the substrate (organic compounds scatter more than the inorganic part).[1]

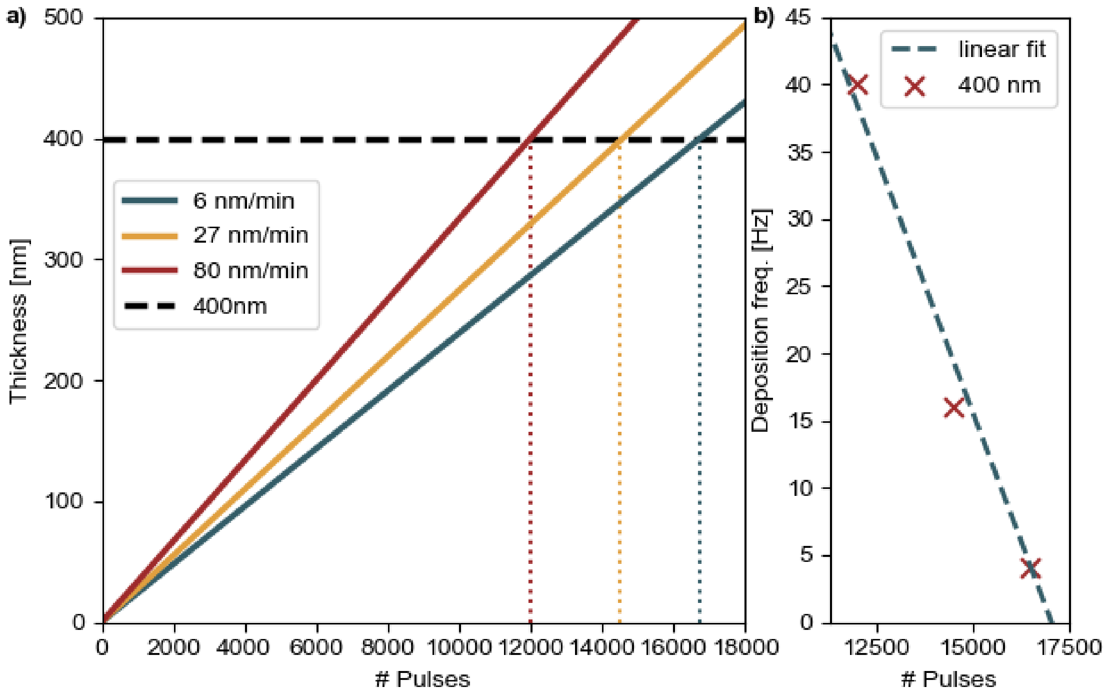

Figure S4: Estimated linear correlation between the film thickness and the number of UV laser pulses applied to the single source target for different deposition frequencies a), b) the number of pulses needed to get 400 nm film for different deposition frequencies.

To ensure film homogeneity, the substrate must complete a full scan in a predefined scanning pattern, which takes approximately 5 minutes per cycle, see illustration in Figure S5. Consequently, the deposition time—dictated by the number of pulses and the UV laser frequency—must be an integer multiple of this scan time (5 minutes). At high deposition rates, this poses a significant challenge, as the deposition time becomes very short, making it difficult to achieve precise control over the desired film thickness. In such cases, a single scan can result in the deposition of several hundred nanometers, limiting the ability to fine-tune

thickness. For 40 Hz laser repetition rate ($\sim$ 80 nm/min growth), the whole film is deposited in just one scan (330 nm).

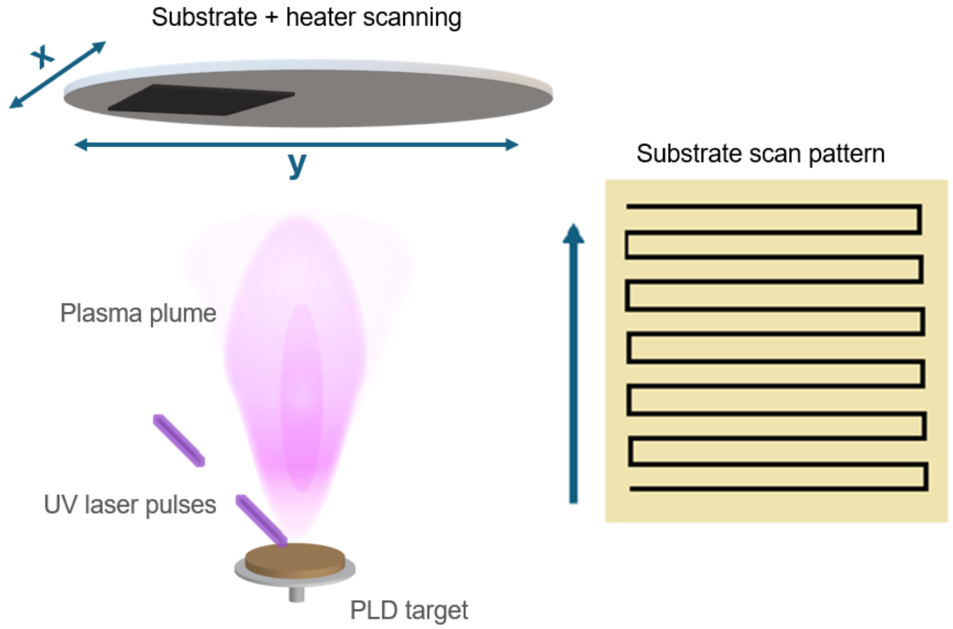

Figure S5: Illustration of the substrate scanning pattern during PLD.

We did test changing the target to a composition that was 50:50 MA:FA instead of the 75:25 MA:FA used throughout the study, however, we didn't show it to avoid confusion and missing the main point of the article. Furthermore, to improve the performance, we added 20 mol% $PbCl_2$ vs PbI_2 to both targets. We added in the SI the PL plot comparing the PL peak position of deposited thin films using different targets (Figure S6). In this plot, we see how the bandgap is shifted towards higher energies when the 75:25 MA:FA target at high frequencies is used compared to the 50:50 MA:FA target at high frequencies where the peak position is close to the low deposition rate results with the standard 75:25 MA:FA target. This means that for future optimization processes, fine-tinning the target composition together with the laser frequency is key.

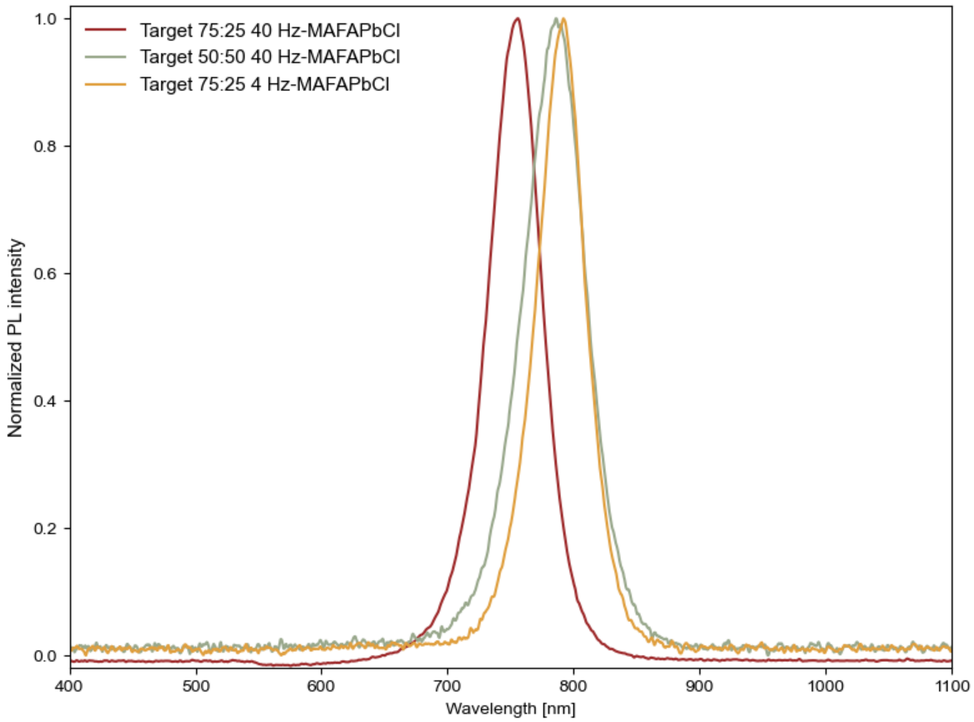

Figure S6: PL comparison of MHP thin films deposited using different target compositions.

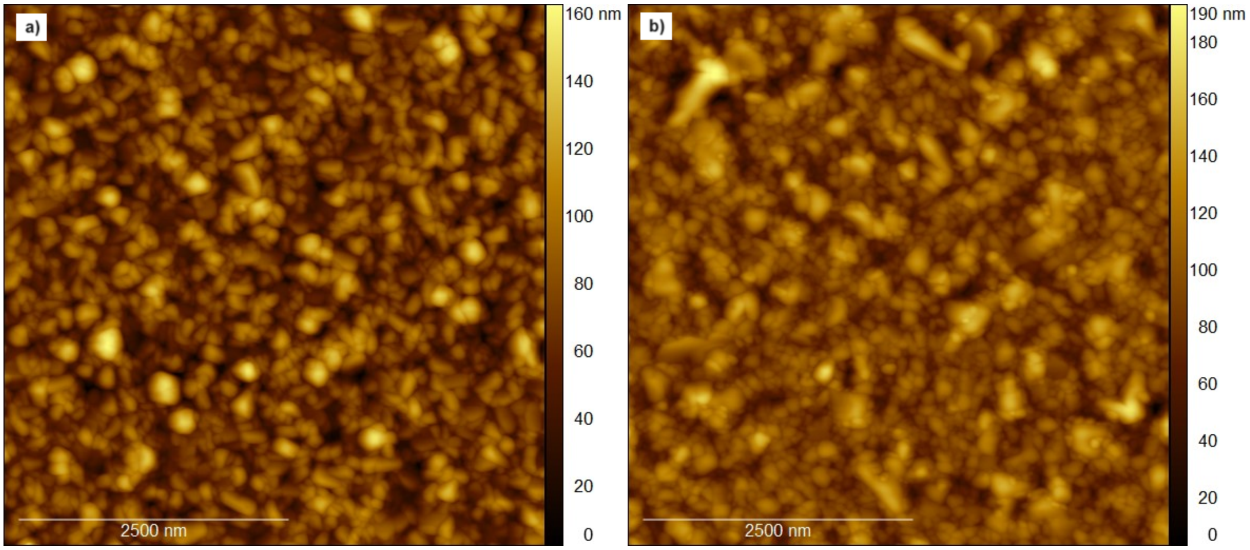

Figure S7: AFM surface topography scan 5×5 μm for different deposition rates, a) 6 nm/min (r_{RSM} = 21 nm), b) 80 nm/min (r_{RSM} = 21 nm).[2]

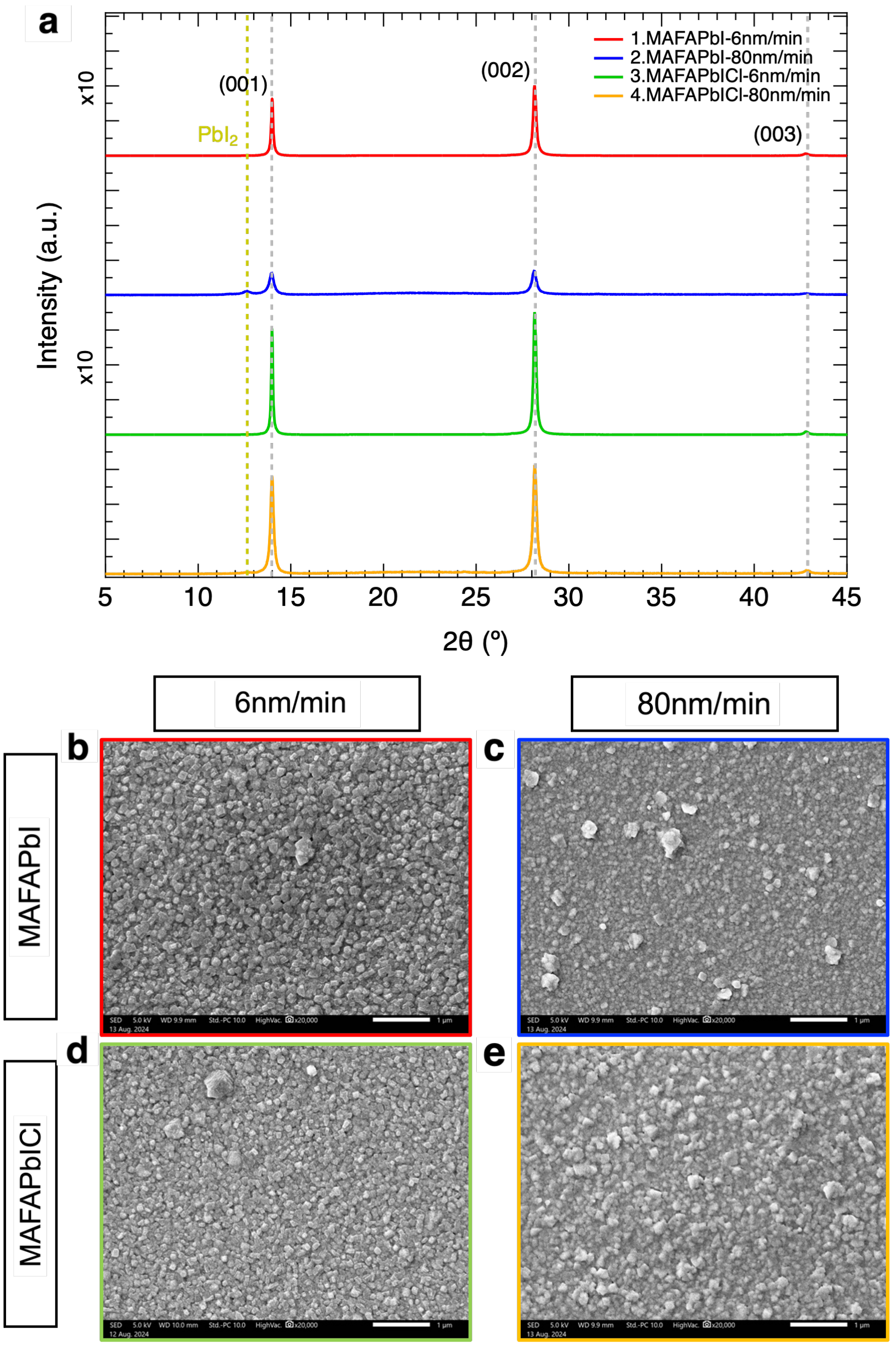

Figure S8: a) XRD patterns and b) top-view SEM images at x20000 magnification of PLD grown $MA_{1-x}FA_xPbI_3$ and $MA_{1-x}A_xPbI/Cl_3$ films with 6 nm/min and c) 80nm/min deposition rates. Note: the XRD patterns are from the films used during the in-situ PL measurements, i.e. the growth is static and interrupted everytime the PL measurement is taken. This results in less random (more oriented) growth at high deposition rates, as compared to the results from Fig. 2a. Moreover, the substrate of these samples is fused silica and not ITO/2PACz. The effect of interrupted growth on final film orientation has been observed previously,[3,4] and will be a subject of further studies for MHPs.

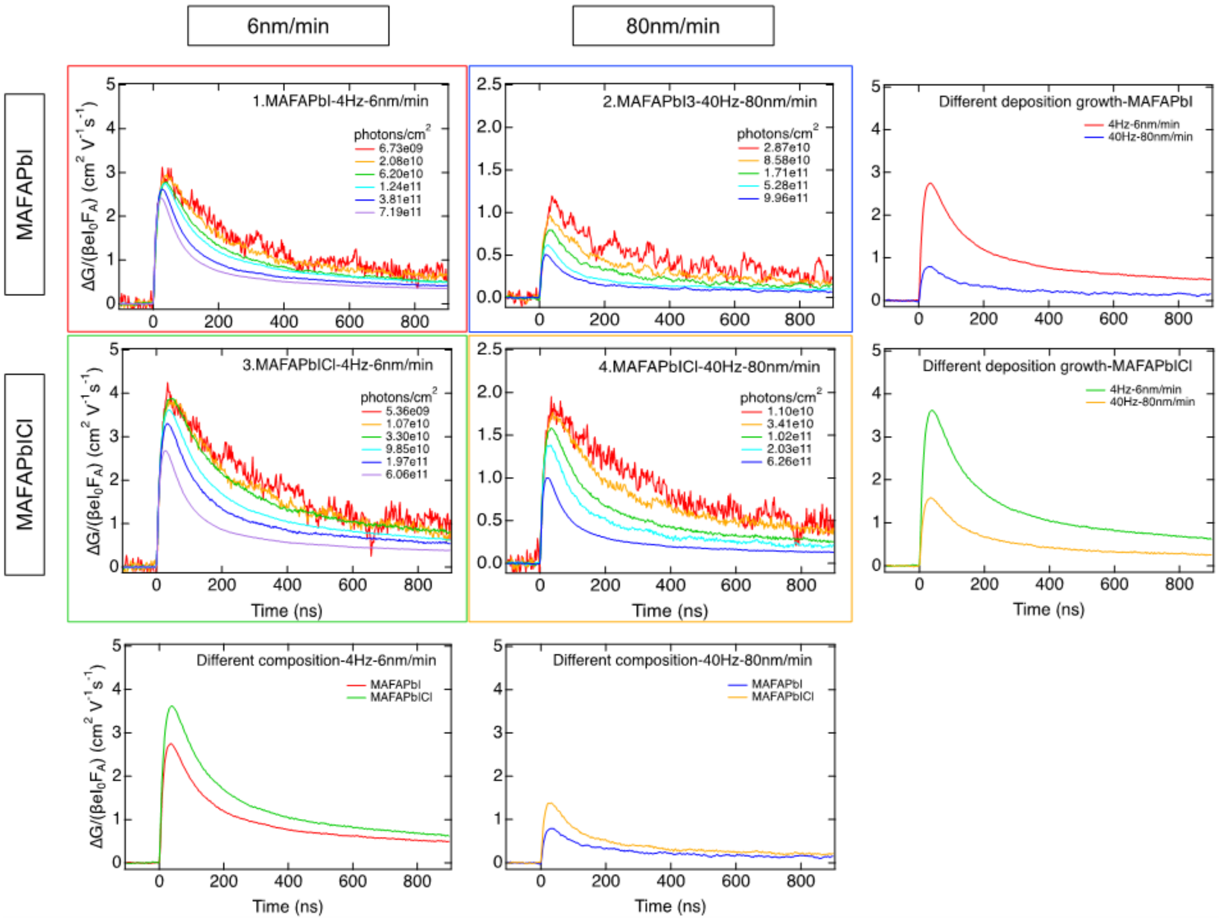

Figure S9: Photoconductance traces for perovskite thin films with varying compositions, namely with (upper row) and without (bottom row) Cl- addition, and with varying growth rates, namely for slow (left column) and fast (right column) PLD deposition. For the comparisons, TRMC traces measured at the same laser intensities ($\approx 1.5 \times 10^{11}$ photons$\cdot$cm^{-2}) and excitation wavelength ($\lambda = 650$ nm) are shown.

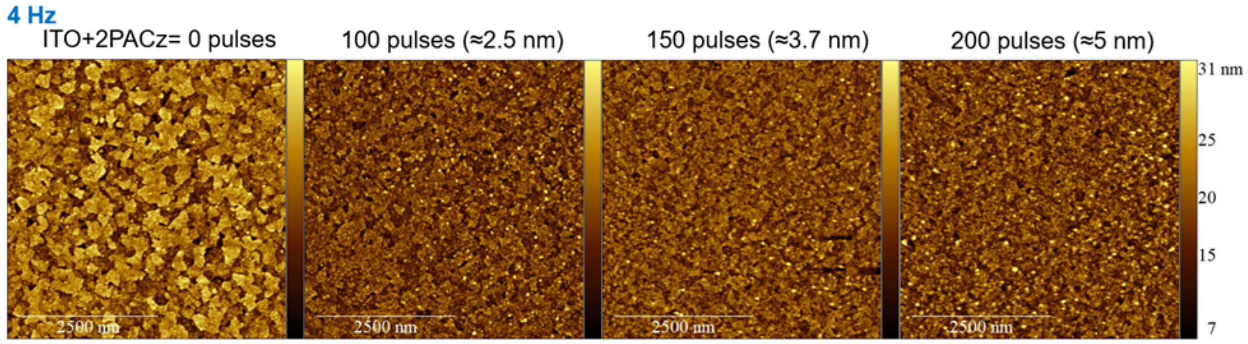

Figure S10: Substrate morphology and initial stages (nuclei) perovskite deposition

The initial stages of perovskite deposition are shown in Figure S10. Then the growth of the MHP thin film transitions into a distinct columnar morphology (the thermodynamical approach is not ideal $\rightarrow$ kinetically driven phenomenon), which we believe cannot be adequately described by the classical thin-film growth models, but the Volmer–Weber would probably be the "closest".

References

(1) Soto-Montero, T.; Soltanpoor, W.; Kralj, S.; Birkhölzer, Y. A.; Remes, Z.; Ledinsky, M.; Rijnders, G.; Morales-Masis, M. Single-source pulsed laser deposition of $MAPbI_3$. 2021 IEEE 48th Photovoltaic Specialists Conference (PVSC). 2021; pp 1318–1323.

(2) Nečas, D.; Klapetek, P. Gwyddion: an open-source software for SPM data analysis. *Central European Journal of Physics* **2012**, *10*, 181–188.

(3) Koster, G.; Rijnders, G. J.; Blank, D. H.; Rogalla, H. Imposed layer-by-layer growth by pulsed laser interval deposition. *Applied physics letters* **1999**, *74*, 3729–3731.

(4) Blank, D. H.; Koster, G.; Rijnders, G. A.; Van Setten, E.; Slycke, P.; Rogalla, H. Epitaxial growth of oxides with pulsed laser interval deposition. *Journal of crystal growth* **2000**, *211*, 98–105.
